# Efficacy and safety of sacubitril/valsartan on heart failure with preserved ejection fraction: A meta-analysis of randomized controlled trials

**DOI:** 10.3389/fcvm.2022.897423

**Published:** 2022-09-08

**Authors:** Wanqian Yu, Hongzhou Zhang, Wen Shen, Fan Luo, Shuai Yang, Lujin Gan, Yuanbin Zhao, Pingping Yang, Qinghua Wu

**Affiliations:** ^1^Department of Cardiovascular Medicine, The Second Affiliated Hospital of Nanchang University, Nanchang, China; ^2^Medical Center of the Graduate School, Nanchang University, Nanchang, China; ^3^Department of Endocrinology and Metabolism, The Second Affiliated Hospital of Nanchang University, Nanchang, China

**Keywords:** sacubitril/valsartan, LCZ696, heart failure, heart failure with preserved ejection fraction (HFpEF), meta-analysis

## Abstract

**Aims:**

The efficacy and safety of sacubitril/valsartan for patients with heart failure with preserved ejection fraction (HFpEF) are controversial. Hence, the primary objective of the study was to evaluate the efficacy and safety of sacubitril/valsartan treatment for patients with HFpEF.

**Methods and results:**

We used the PubMed, Embase, and Web of Science databases to search for randomized controlled trials of sacubitril–valsartan in patients with HFpEF. Three studies, involving a total of 7,663 patients, were eligible for inclusion. Sacubitril–valsartan reduced the risk of hospitalization for heart failure (HF) [odds ratio (*OR*): 0.78; 95% *CI*: 0.70–0.88; *p* < 0.0001] and the incidence of worsening renal function [risk ratio (*RR*): 0.79, *p* = 0.002] among patients with HFpEF in the three trials, but there was no significant reduction in all-cause mortality (0.99, 95% *CI*: 0.84–1.15; *p* = 0.86) or cardiovascular mortality (0.95, 95% *CI*: 0.78–1.15; *p* = 0.16). Moreover, sacubitril/valsartan was associated with an increased risk of symptomatic hypotension (*RR*: 1.44; *p* < 0.00001) and angioedema (*RR*: 2.66; *p* < 0.04); there was no difference for decreasing the incidence of hyperkalemia (*RR*: 0.89; *p* = 0.11).

**Conclusion:**

Compared with valsartan or individualized medical therapy (IMT), sacubitril/valsartan significantly decreased the risk of hospitalization for HF and reduced the incidence of renal dysfunction.

## Introduction

Heart failure (HF) is a clinical syndrome caused by structural or functional cardiac abnormalities and manifests as an increase in internal pressure or a decrease in cardiac output. Due to increases in the size of the aging population and the incidence of risk factors, the prevalence of heart failure has been on the rise as well (1). To date, almost half of the 5 million patients with heart failure in the United States display heart failure with preserved ejection fraction (HFpEF) (2). The pathophysiological mechanism of HFpEF includes left ventricular (LV) structure and remodeling, LV diastolic limitations, and LV systolic limitations. Currently, however, no effective treatment drug exists (3).

Sacubitril/valsartan is the first new angiotensin receptor neprilysin inhibitor for treating hypertension and heart failure (4); such drugs treat HFpEF by blocking a profibrotic/prohypertrophic mechanism and stimulating an antifibrotic/antihypertrophic mechanism. Compared with angiotensin-converting enzyme inhibitors (ACEIs) or angiotensin receptor blockers (ARBs), sacubitril/valsartan can increase the levels of many vasoactive peptides, especially natriuretic peptides (NPs), which have powerful effects on sodium, fluid balance, and vascular diastolic function by inhibiting the renin–angiotensin–aldosterone system (RAAS), reducing the sympathetic nervous system activity, and exerting antiproliferative and anti-muscle hypertrophy effects (5).

Kuno et al. (6) demonstrated that treatment with sacubitril/valsartan can reduce the risk of hospitalization for HF, but the results of the PARAGON-HF trial did not reach a similar conclusion (7). The use of sacubitril/valsartan for patients with HFpEF is still controversial. Thus, we conducted a systematic review to evaluate the efficacy and safety of sacubitril/valsartan treatment for patients with HFpEF.

## Methods

### Protocol registration

We registered the protocol for this systematic review with the International Prospective Register of Systematic Reviews (PROSPERO) (CRD42020207370).

### Data sources and search strategy

We searched for articles in the PubMed, Embase, and Web of Science databases through 13 December 2021 using the following search terms: “heart failure with preserved ejection fraction” or “heart failure with normal ejection fraction” or “diastolic heart failure” or “diastolic dysfunction” or “preserved cardiac function heart failure” or “HFpEF” and “sacubitril valsartan” or “sacubitril/valsartan” or “lcz696” or “sacubitril” or “entresto” In addition, we reviewed the corresponding reference lists of the retrieved articles to avoid missing any relevant studies. The meta-analysis was conducted and reported according to the Preferred Reporting Items for Systematic Reviews and Meta-Analyses (PRISMA) guidelines (8).

### Selection criteria

Eligible studies had to meet the following inclusion criteria: (1) the enrolled participants had HFpEF (LVEF ≥ 45%); (2) the study design was a randomized controlled trial (RCT) of the treatment group (sacubitril/valsartan) and the control group; and (3) the trial provided primary outcome data (cardiovascular mortality, all-cause mortality, hospitalization for HF, and main adverse events, such as symptomatic hypotension, worsening renal function, hyperkalemia, and angioedema).

The exclusion criteria were as follows: (1) duplicated trials; (2) studies, such as systematic reviews, comments, case reports, conference abstracts, and editorials; and (3) RCTs that did not involve humans. The details are shown in Table 1.

**Table 1:** Inclusion and exclusion criteria.

**Category**	**Inclusion criteria**	**Exclusion criteria**
Patient population	HFpEF defined as LVEF ≥ 45%	Not HFpEF
Intervention/comparator	Sacubitril/valsartan and control group	Other drugs vs. control group
Outcome	“All-cause mortality”, “cardiovascular causes”, “hospitalization for HF”, “symptomatic hypotension”, “worsening renal function”, “hyperkalemia”, “angioedema”	No “all-cause mortality”, “cardiovascular causes”, “hospitalization for HF”, “symptomatic hypotension”, “worsening renal function”, “hyperkalemia”, and “angioedema” outcomes reported
Study design	RCT	Not-RCTs: systemic reviews, comments, case reports, conference abstracts, editorials, and not in human
Language	English	Non-English language publications

HFpEF, heart failure with preserved ejection fraction; HF, heart failure; LVEF, left ventricular ejection fraction; RCT, randomized controlled trial.

### Data extraction and quality assessment

YW and ZH independently extracted data and assessed the quality of the studies from the electronic database. The relevant data we extracted included the following: the baseline characteristics of the trials, interventions, comparisons, sample size, the medication used, and follow-up duration. The outcomes included death from any cause, death from cardiovascular causes, hospitalization for HF, symptomatic hypotension, renal dysfunction, hyperkalemia, and angioedema. Disagreements were resolved by discussion with a third author (W. Q. H.).

### Risk of bias assessment

The methodological quality of the three included RCTs was assessed by using the Cochrane Collaboration Risk of Bias Tool (Review Manager 5.4.1), which included the following sections: selection, performance, detection, attrition, reporting, and other biases. The results are shown in Supplementary Figure S1.

### Statistical analysis

Review Manager Version 5.4.1 was used to analyze the data. The efficacy and safety outcomes were measured as dichotomous outcome variables and compared between the sacubitril–valsartan group and the control group. The pooled odds ratio (*OR*) or risk ratio (*RR*) and the corresponding 95% confidence interval (*CI*) were calculated in the comparative analyses. We assessed heterogeneity by using the *I*^2^ test and Cochran's χ^2^ test. The total variation in the studies was described by the *I*^2^ statistic, which reflected heterogeneity. When the heterogeneity test result was *I*^2^ < 50% and *p* > 0.10, we used a fixed-effects model for data analysis; *I*^2^ > 50% or a corresponding *p-*value < 0.10 indicated statistical heterogeneity among the studies that needed further analysis. All *p*-values were two-tailed, with statistical significance indicated at 0.05 and *CI*s reported at the 95% level. When *I*^2^ was >45%, a sensitivity analysis was further performed by sequentially deleting each study and reanalyzing the datasets of all remaining studies.

## Results

### Description of the study selection process and study characteristics

**Figure 1:**
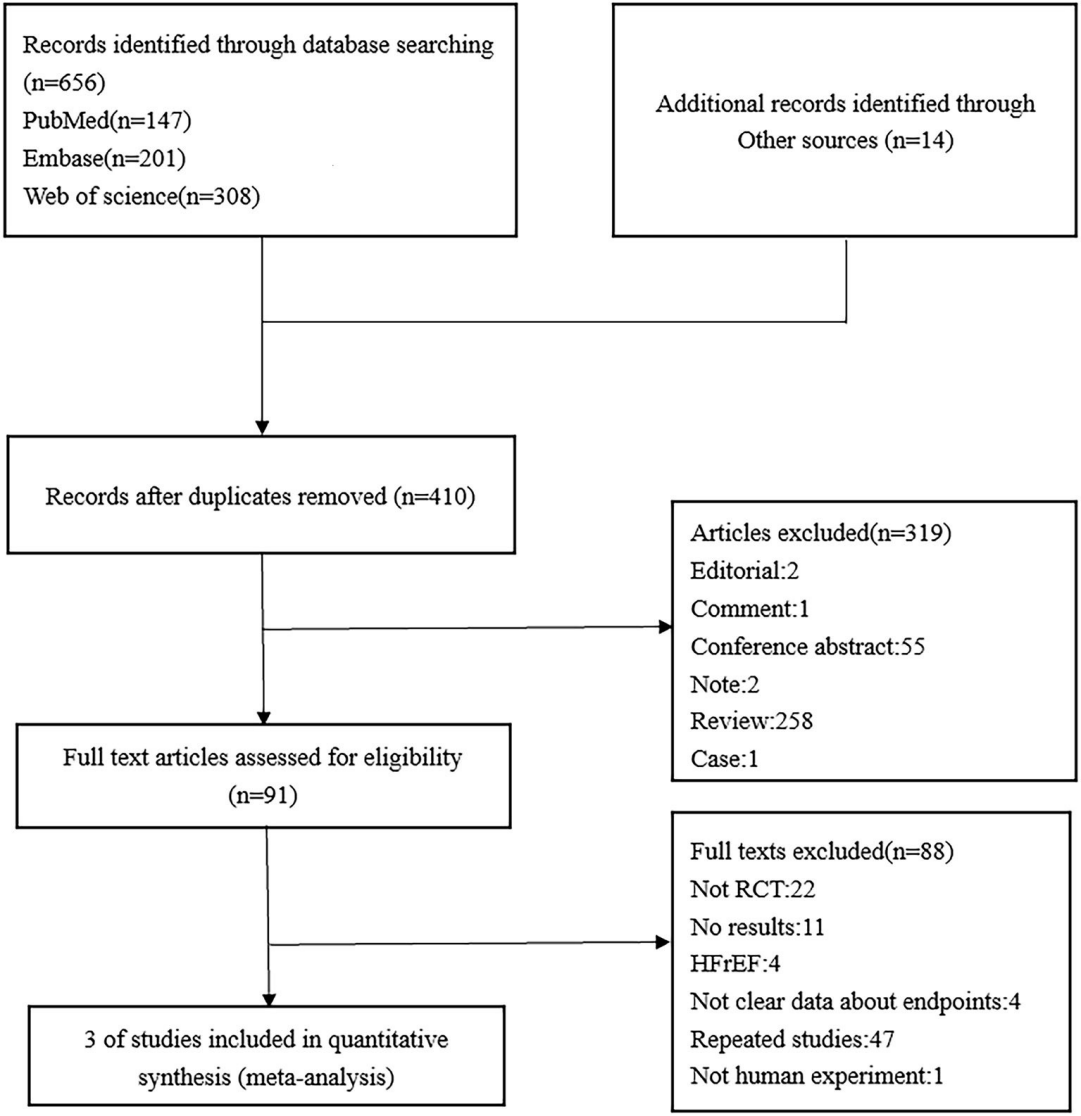
The preferred reporting items for systematic reviews and meta-analyses (PRISMA) diagram of the study selection process for the meta-analysis.

A detailed flowchart of the study selection is presented in Figure 1. Ultimately, three double-blind RCTs that involved a total of 7,663 patients were included in our study (Figure 1). The baseline characteristics of the included studies are shown in Table 2 and include follow-up duration, left ventricular ejection fraction (LVEF), primary efficacy outcomes, and key adverse events.

**Table 2:** Baseline characteristics of RCTs.

	**PARALLAX-HF Pieske 2021**		**PARAGON-HF Solomon 2019**		**PARAMOUNT Solomon 2012**	
Group	Sac/Val (*n* = 1,281)	IMT (*n* = 1,285)	Sac/Val (*n* = 2,407)	Valsartan (*n* = 2,389)	Sac/Val (*n* = 149)	Valsartan (*n* = 152)
Follow-up duration	12 weeks		8 months		36 weeks	
Age, years	72.9 ± 8.4	72.4 ± 8.6	72.7 ± 8.3	72.8 ± 8.5	70.9 ± 9.4	71.2 ± 8.9
Female (%)	638 (49.8)	627 (48.8)	1,241 (51.6)	1,238 (51.8)	85 (57)	85 (56)
White race (%)	1,112 (86.8)	1,117 (86.9)	1,963 (81.6)	1,944 (81.4)	NA	NA
**NYHA class (%)**						
I	1 (0.1)	4 (0.3)	73 (3.0)	64 (2.7)	1 (1)	1 (1)
II	858 (67)	876 (68.2)	1,866 (77.5)	1,840 (77.0)	120 (81)	119 (78)
III	416 (32.5)	401 (31.2)	458 (19.0)	474 (19.8)	28 (19)	32 (21)
IV	5 (0.4)	4 (0.3)	8 (0.3)	11 (0.5)		
LVEF (%)	56.7 ± 8.3	56.2 ± 8.0	57.6 ± 7.8	57.5 ± 8.0	58 ± 7.3	58 ± 8.1
Heart rate, beats/min	NA	NA	70.6 ± 12.3	70.3 ± 12.2	69 ± 12	70 ± 14
Systolic blood pressure, mmHg	132.6 ± 13.9	134.2 ± 14.5	130.5 ± 15.6	130.6 ± 15.3	137.1 ± 11.2^*^	135.7 ± 14.2^*^
Body mass index	30.6 ± 5.0	30.5 ± 4.8	30.2 ± 4.9	30.3 ± 5.1	30.1 ± 5.5	29.8 ± 6.1
Scr, mg/dl			1.1 ± 0.3	1.1 ± 0.3		
GFR, ml/min/1.73 m^2^	62.5 ± 20.2	62.7 ± 19.6	63 ± 19	62 ± 19	67 ± 19.4	64 ± 21.3
Potassium, mmol/L	NA	NA	NA	NA	NA	NA
**Medications at baseline (%)**						
ACE inhibitors	1,115 (87.1)	1,124 (87.5)	2,074 (86.2)	2,065 (86.4)	83 (56)	80 (53)
ARBs					57 (38)	62 (41)
Diuretics	1,277 (99.8)	1,282 (99.8)	2,294 (95.3)	2,291 (95.9)	149 (100)	152 (100)
Beta-blockers	1,071 (83.7)	1,066 (83)	1,922 (79.9)	1,899 (79.5)	117 (79)	121 (80)
Aldosterone antagonists	419 (32.7)	392 (30.5)	592 (24.6)	647 (27.1)	28 (19)	35 (23)
SGLT-2 inhibitors	34 (2.7)	26 (2.0)	NA	NA	NA	NA

Sac/Val, sacubitril–valsartan; NYHA, New York Heart Association; LVEF, left ventricular ejection fraction; Scr, serum creatinine; GFR, glomerular filtration rate; ACE, angiotensin-converting enzyme; ARBs, angiotensin receptor blockers; RCTs, randomized controlled trials.

* The sample mean and standard deviation (SD) were estimated from the sample size, median, and interquartile range (IQR) through the special website (http://www.math.hkbu.edu.hk/~tongt/papers/median2mean.html).

### Primary efficacy outcomes

**Figure 2:**
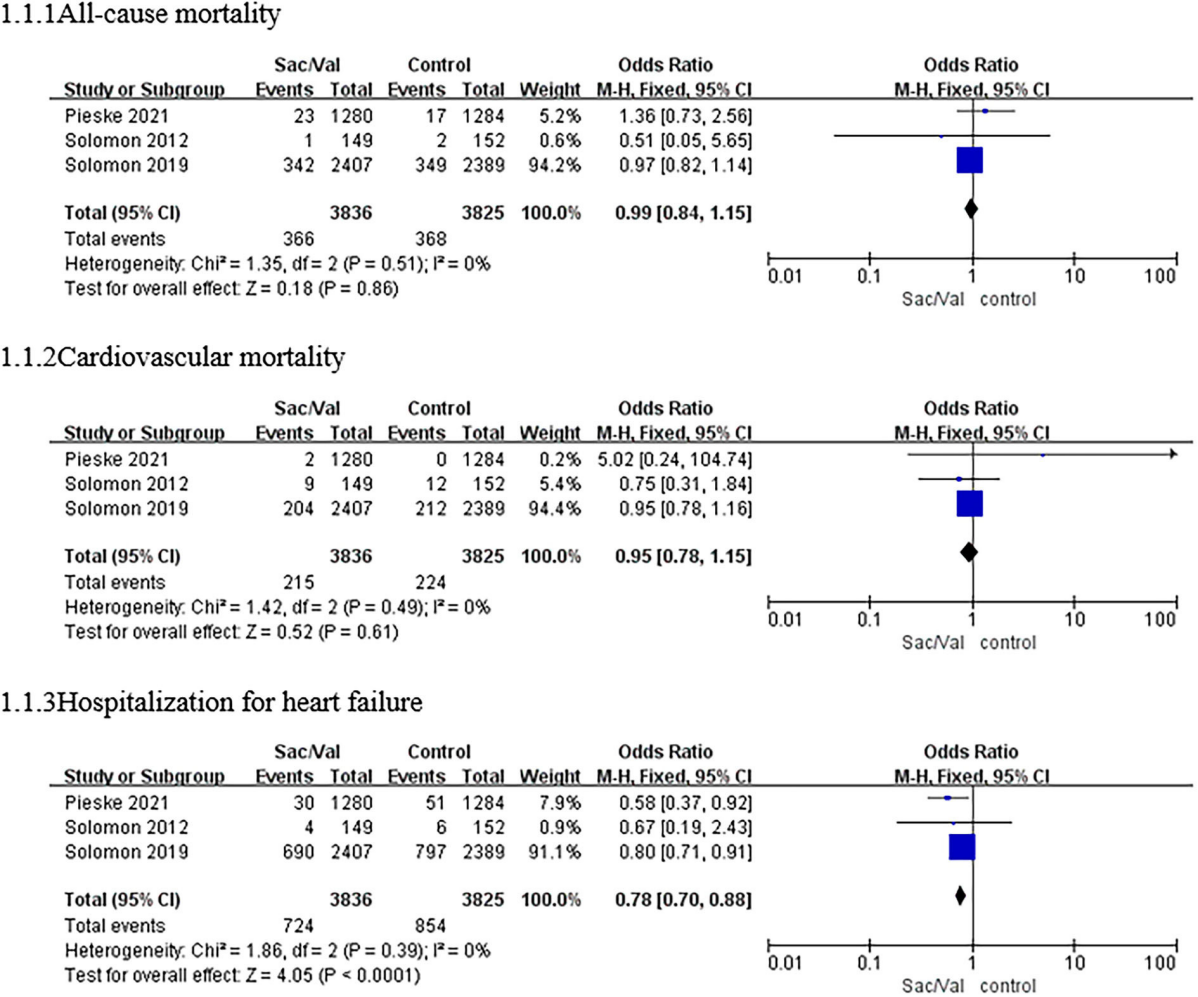
A forest plot of the effective outcomes of all-cause mortality, cardiovascular mortality, and hospitalization for HF in different patients with HFpEF. HF, heart failure; HFpEF, heart failure with preserved ejection fraction; Sac/Val, sacubitril/valsartan.

All three trials included in the meta-analysis reported the primary outcome. The estimated results of the primary efficacy outcomes of death from all causes, death from cardiovascular causes, and hospitalization for HF are presented in Figure 2.

The heterogeneity test results showed no significant heterogeneity among the three studies (*p* = 0.51, *I*^2^ = 0%); thus, the meta-analysis was carried out using a fixed-effects model. Regarding the outcome of all-cause mortality, the pooled *OR* based on three studies was 0.99 (95% *CI*: 0.84–1.15, *p* = 0.86). The *OR* of cardiovascular mortality based on three studies was 0.95 (95% *CI*: 0.78–1.15, *p* = 0.16; *p* = 0.49 for heterogeneity, *I*^2^ = 0%). There were no significant differences in all-cause mortality or cardiovascular mortality among the patients with HFpEF between the sacubitril–valsartan group and the control group (Figure 2).

For the risk of hospitalization for HF, no significant heterogeneity was observed among the three studies (*p* = 0.39, *I*^2^ = 0%), and a fixed-effects model was used. Compared with valsartan or individualized medical therapy (IMT), sacubitril–valsartan reduced the composite risk of hospitalization for HF by 22% based on the three studies, and the pooled *OR* was 0.78 (95% *CI*: 0.70–0.88, *p* < 0.0001; Figure 2).

### Adverse events of interest

#### Symptomatic hypotension

**Figure 3:**
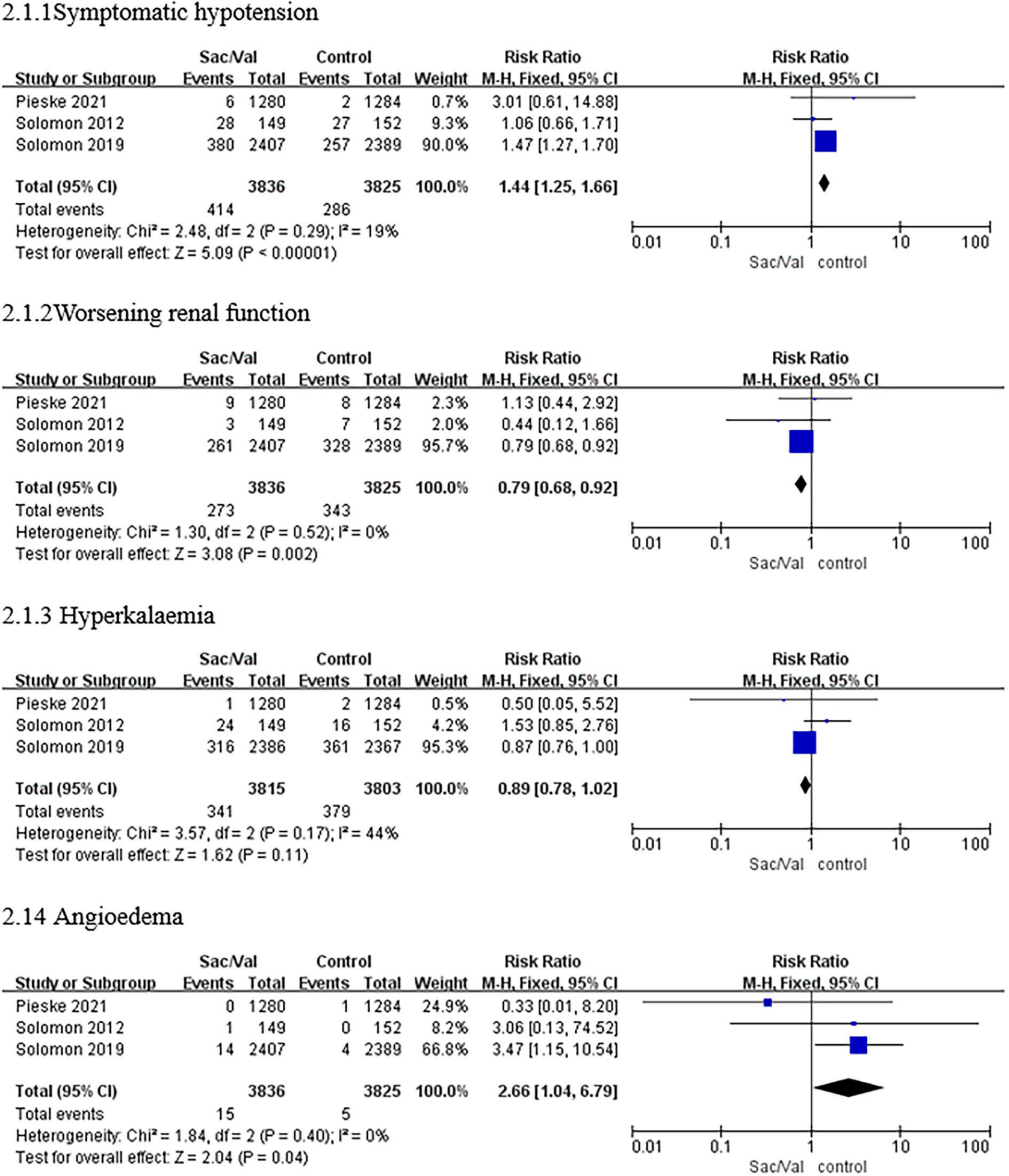
A forest plot of the safety outcomes of symptomatic hypotension, worsening renal function, hyperkalemia (≥5.5 mmol/L), and angioedema. Worsening renal function was defined as a decrease in estimated glomerular filtration rate (eGFR) ≥35% or an increase in serum creatinine ≥0.5 mg/dl from baseline and a decrease in eGFR ≥25% from baseline or serum creatinine >2.5 mg/dl. Sac/Val, sacubitril/valsartan; eGFR, estimated glomerular filtration rate.

Regarding this adverse event, compared with valsartan or IMT, sacubitril–valsartan led to a higher risk of symptomatic hypotension in all three trials with a pooled *RR* of 1.44 (95% *CI*: 1.25–1.66, *p* < 0.00001; *p* = 0.29 for heterogeneity, *I*^2^ = 19%; Figure 3).

#### Worsening renal function

As shown in Figure 3, the treatment with sacubitril–valsartan was related to a significant reduction in the incidence of worsening renal function with a pooled *RR* of 0.79 (95% *CI*: 0.68–0.92, *p* = 0.002; *p* = 0.52 for heterogeneity, *I*^2^ = 0%).

#### Hyperkalemia

Regarding hyperkalemia, there was no significant heterogeneity in the incidence between the sacubitril/valsartan group and the control group in all trials (*p* = 0.17, *I*^2^ = 44%), as shown in Figure 3. Furthermore, sacubitril–valsartan led to a numerically higher risk of hyperkalemia than valsartan or IMT with a pooled *RR* of 0.89 (95% *CI*: 0.78–1.02, *p* = 0.11; Figure 3).

#### Angioedema

The results showed that patients receiving sacubitril/valsartan had a higher risk of angioedema in all three trials, with a pooled *RR* of 2.66 (*CI:* 1.04–6.79, *p* < 0.04; *p* = 0.40 for heterogeneity, *I*^2^ = 0%; Figure 3).

## Discussion

The present meta-analysis, which involved 7,663 patients, is the first to provide composite evidence of the efficacy and safety of sacubitril/valsartan in patients with HFpEF by pooling data from relevant RCTs. All of the studies included in this meta-analysis were randomized, controlled, and double-blind multicenter clinical trials. The results suggest that compared with valsartan or IMT, sacubitril/valsartan showed a significant advantage in reducing the rate of hospitalization for hazard ratio (*HR*). However, there was no obvious difference in the reduction in all-cause mortality or in the rate of death from cardiovascular diseases. Sacubitril/valsartan increased the risk of hypotension and angioedema, but it could reduce the incidence of worsening renal function. At the same time, the occurrence of hyperkalemia was numerically higher, but not statistically significant, in the sacubitril/valsartan group than in the control group.

The PARAMOUNT study (9) was a prospective study that compared the treatment of patients with HFpEF. The results showed that the level of N-terminal pro-brain natriuretic peptide (NT-proBNP), a key biomarker used in HF diagnosis to reflect the severity of heart failure (10, 11), in patients with HFpEF was significantly lower in the treatment group than in the valsartan group after 12 weeks of treatment (12). After 36 weeks of treatment, the left atrial volume in the treatment group was significantly lower than that in the control group, and the cardiac function in the treatment group was significantly better than that in the control group. Notably, the left atrial volume and dimension were significantly reduced in patients with HFpEF. The PARALLAX trial had similar results at 12 weeks (ratio 0.84, 95% *CI:* 0.80–0.88, *p* < 0.0001) (13). Left atrial volume is a biomarker of cardiac diastolic function (14). In addition, the Kansas City Cardiopathy Questionnaire (KCCQ) score was significantly lower than that of the control group. Studies have confirmed that sacubitril/valsartan has a certain therapeutic effect on patients with HFpEF and is beneficial for inhibiting ventricular remodeling.

The PARAGON-HF was a randomized, double-blind, and active-controlled trial (15). The results of the PARAGON trial (7) did not indicate a statistically significant difference but revealed that compared with valsartan, sacubitril/valsartan reduced the risk of experiencing the major endpoints (cardiovascular death and total hospitalization for HF) by 13% (*RR*: 0.87, 95% *CI*: 0.75–1.01, *p* = 0.06). Notably, the subgroup analysis in this trial showed that sacubitril/valsartan had a beneficial effect on patients with an LVEF between 45 and 57% (*RR*: 0.78, 95% *CI*: 0.64–0.95). This could be because these patients have systolic dysfunction, and sacubitril/valsartan has the same physiologic effects in these patients as it does in those with heart failure with reduced ejection fraction (HFrEF). For the secondary endpoints, sacubitril/valsartan significantly improved the New York Heart Association (NYHA) grade by 45% compared with valsartan (*OR*: 1.45, 95% *CI*: 1.13–1.86). Similarly, compared with the valsartan group, the sacubitril/valsartan group had a higher percentage of patients with a KCCQ clinical summary score of 5 or higher (33.0 vs. 29.6%). In addition, the incidence of the compound renal endpoint in the sacubitril/valsartan group was significantly reduced compared with that in the valsartan group (*HR*: 0.50, 95% *CI*: 0.33–0.77). The reasons for the negative results of the PARAGON trial (7) may be as follows: most patients were treated with ACEIs or ARBs before participating in the trial or the main endpoints of observation were different. Another study showed that the occurrence of HFpEF is heterogeneous, and this result was consistent with the PARAGON trial (12). In the PARAGON trial (7), the subgroup analysis showed that the two pre-specified subgroups—those with LVEF ≤ 57% and women—received significant benefits: the risk of the primary endpoints decreased by 27% in women and by 22% in the low LVEF group (16), demonstrating that sacubitril/valsartan may benefit high-risk patients with HFpEF (17). Based on these results, patients with HFpEF with structural heart disease and volume overload may be more sensitive to the sacubitril/valsartan treatment. In the PARALLAX trial (13), hospitalization for HF or the compound endpoint of all-cause mortality or heart failure for hospitalization was lower in the sacubitril–valsartan group than in the IMT group. In the PARALLAX trial (13), sacubitril/valsartan did not show superiority over valsartan in terms of the KCCQ score (13), which may be related to the severity of the patient's condition and insufficient treatment time.

In the PARAGON-HF study (7) and the PARALLAX study (18, 19), there was a higher risk of symptomatic hypotension caused by sacubitril/valsartan. This result is consistent with this meta-analysis; sacubitril/valsartan increased the incidence of hypotension with an *RR* of 2.66 (*p* = 0.04) compared with enalapril or valsartan. This finding coincides with previous studies (20–22). In the TITRATION study (23), patients with lower systolic blood pressure were successfully treated by gradual titration, which suggested that patients with lower systolic blood pressure can also use sacubitril/valsartan. Although hypotension can cause insufficient blood perfusion in the kidneys, causing kidney damage, previous studies (24, 25) found that sacubitril/valsartan could protect renal function, which was similar to our outcome that treatment with sacubitril/valsartan could protect renal function to prevent deterioration. Rubattu found that sacubitril/valsartan was more effective in reducing cardiovascular risk in rats with chronic kidney disease than valsartan alone (26). The UK HARP-III experiment showed that sacubitril/valsartan and irbesartan had similar effects on renal function after 12 months of follow-up (27). Damman conducted further studies that had similar results (28). Moreover, the results of the PARAMOUNT (9) and PARAGON-HF (7) studies showed that patients in the sacubitril/valsartan group had more beneficial effects on kidney function than those in the valsartan (ARB) group. The main mechanisms of renal protection by sacubitril/valsartan are as follows: (A) direct action on the kidney, inhibiting water and sodium reabsorption in the proximal and distal nephrons (the inhibition of proximal sodium and potassium exchange, distal sodium chloride exchange, and sodium channels in the collecting tubule) (29); (B) indirectly inhibits the release or action of other vasoconstrictors (renin, vasopressin, aldosterone, and norepinephrine), resulting in natriuretic and diuretic effects (30); and (C) inhibits inflammation and oxidative stress, delays glomerulosclerosis, etc (31, 32). Consequently, sacubitril/valsartan has more beneficial effects on renal function than ACEIs or ARBs in patients with HF. Concerning the incidence of hyperkalemia, there was no difference between the two groups (95% *CI*: 0.78–1.02, *p* = 0.11), which was consistent with previous studies (33). Although the risk of angioedema was low in the included studies, the pooled analysis indicated that sacubitril/valsartan led to a higher risk of angioedema (*CI*: 1.04–6.79, *p* < 0.04). However, the occurrence of angioedema is known to be related to the inhibition of bradykinin degradation (34), and sacubitril/valsartan does not inhibit ACE or aminopeptidase P, so it does not increase the risk of angioedema (4, 35). The reason for the result needs further research.

Of note, although no medical therapy has been shown to reduce all-cause or cardiovascular death in HFpEF trials, according to the latest AHA/ACC/HFSA heart failure guidelines (2022) (36), a number of drugs other than sacubitril/valsartan are recommended for treating HFpEF. Despite a lack of strong evidence, diuretics have been used in HFpEF to reduce symptoms due to volume overload (37). Renin–angiotensin–aldosterone system (RAAS) inhibitors and mineralocorticoid receptor antagonists have a well-established role in HFrEF but have been less effective in HFpEF, possibly because the RAAS plays a less prominent pathophysiological role as LVEF increases (38). Trials of ACEI and ARB use in HFpEF have not shown a significant reduction in all-cause or cardiovascular death, but these drugs may have reduced the risk of HF hospitalization (39–41). The TOPCAT trial (42) found no overall benefit in the primary composite outcome of cardiovascular death or hospitalization for heart failure. However, spironolactone reduced the risk of HF hospitalization in both the TOPCAT and TOPCAT-Americas subgroups, which could be linked to improved diastolic function in patients with HFpEF. In the EMPEROR-Preserved trial (43), a sodium-glucose cotransporter-2 inhibitor (SGLT-2i; empagliflozin) reduced the risk of composite cardiovascular death or total HF hospitalization by 21% in patients with HF with LVEF > 40%, driven primarily by a significant reduction in the HF hospitalization of 29% (no significant reduction in cardiovascular death [hazard ratio (*HR*), 0.91; 95% *CI*: 0.76–1.0]), with no benefit for all-cause mortality. In addition, the SOLOIST-WHF trial (44) showed that both patients with HFpEF and HFrEF had a reduced risk of endpoint events (cardiovascular death and HF hospitalizations; LVEF < 50% subgroup: *HR* = 0.72; LVEF ≥ 50% subgroup: *HR* = 0.48), which was driven by the reduction in HF hospitalizations. Sacubitril/valsartan and empagliflozin, two new drugs in the field of heart failure, have some similarities and differences in the clinical trials associated with them (Table 3). In the EMPEROR-Preserved subgroup analysis, patients with LVEF > 60% received no benefit. This result was similar to that in the subgroup of patients with LVEF > 57% in the PARAGON trial (*RR*, 1.00; 95% *CI*: 0.81–1.23). In terms of safety outcome events, while both drugs resulted in symptomatic hypotension, patients taking empagliflozin were more prone to volume depletion and urinary tract infections (45), while those taking sacubitril/valsartan were more susceptible to hyperkalemia and angioedema. Therefore, when assessing cardiac function and making treatment decisions for patients with heart failure, we should not simply use LVEF as the reference indicator; rather, treatment should be individualized.

**Table 3:** The difference between the end-point of the PARAGON trial and of SGLT2i trials.

**Outcome**	**PARAGON-HF 2019 (sacubitril/valsartan)**	**EMPEROR-preserved 2021 (Empagliflozin)**	**SOLOIST-WHF 2021 (Sotagliflozin)**
Total hospitalizations for heart failure and death from cardiovascular causes	RR, 0.87 (0.75–1.01)	HR, 0.79 (0.69, 0.9)	HR, 0.67 (0.52, 0.85)
Hospitalizations for heart failure	RR, 0.85 (0.72–1.00)	HR, 0.71 (0.60, 0.83)	HR, 0.64 (0.49, 0.83)
Death from cardiovascular causes	HR, 0.95 (0.79–1.16)	HR, 0.91 (0.76, 1.09)	HR, 0.84 (0.58, 1.22)
Death from any cause	HR, 0.97 (0.84–1.13)	HR, 1.00 (0.87, 1.15)	HR, 0.82 (0.59, 1.14)

## Limitations

There were some limitations to our study. First, because only three RCTs were included in our work, the sample size of this systematic review was too small, and we could not produce a funnel plot. Second, HFpEF was defined as LVEF ≥ 50%, but the studies included in our work defined HFpEF as LVEF ≥ 45%. Third, unpublished data or articles published not in English were excluded. Fourth, we used data that were not published but were available on ClinicalTrials.gov. Fifth, for meaningful conclusions from the meta-analysis of randomized trials, we used secondary outcomes, not primary outcomes. Sixth, there were many mixed factors that may have caused bias. For example, the comparator drug was different among the three trials included in our work. Finally, the majority of the sample in our work was from the PARAGON-HF trial, which may lead to sample imbalance and affect the results of our work. Thus, further studies are needed to assess more potential clinical benefits of sacubitril/valsartan in patients with HFpEF.

## Conclusion

In conclusion, the existing evidence shows that sacubitril/valsartan not only is effective in the treatment of heart failure but also has a protective effect on kidney function. Although there was no significant reduction in all-cause mortality or cardiovascular mortality, patients treated with sacubitril/valsartan could obtain the benefits of a reduction in hospitalizations for HF and prevention of renal functional deterioration. Moreover, compared with valsartan or IMT, sacubitril/valsartan could increase the risk of symptomatic hypotension and angioedema but did not differ in the elevation of serum potassium. Compared with ACEIs or ARBs, sacubitril/valsartan is a better choice, but it is best to monitor blood pressure and renal function during treatment.

## Data availability statement

The original contributions presented in the study are included in the article/Supplementary material, further inquiries can be directed to the corresponding authors.

## Ethics statement

Ethical review and approval was not required for the study on human participants in accordance with the local legislation and institutional requirements. The patients/participants provided their written informed consent to participate in this study.

## Author contributions

YW and ZH reviewed the articles, performed the meta-analysis and wrote the manuscript. SW, LF, and YS were responsible for the statistical analysis. ZY provided editing assistance. YP and WQ designed and revised the manuscript. All authors have reviewed and agreed on this information before submission.

## Funding

This work was supported by the Provincial Plans-Social Development Areas—Major Projects of Jiangxi Province (No. 20161ACG70012) and the incubation project of the National Natural Science Foundation of the Second Affiliated Hospital of Nanchang University (No. 2021YNFY2021).

## Conflict of interest

The authors declare that the research was conducted in the absence of any commercial or financial relationships that could be construed as a potential conflict of interest.

## Publisher's note

All claims expressed in this article are solely those of the authors and do not necessarily represent those of their affiliated organizations, or those of the publisher, the editors and the reviewers. Any product that may be evaluated in this article, or claim that may be made by its manufacturer, is not guaranteed or endorsed by the publisher.

## Abbreviations

RCTs: randomized controlled trials
HFpEF: heart failure with preserved ejection fraction
HFrEF: heart failure with reduced ejection fraction
HF: heart failure
LV: left ventricular
NPs: natriuretic peptides
RAAS: renin–angiotensin–aldosterone system
ACEI: angiotensin-converting enzyme inhibitor
ARB: angiotensin receptor blocker
IMT: individualized medical therapy
LVEF: left ventricular ejection fraction
OR: odds ratio
RR: risk ratio
HR: hazard ratio
Cl: confidence interval
NT-pro BNP: N-terminal pro-brain natriuretic peptide
NYHA: New York Heart Association
KCCQ: The Kansas City Cardiomyopathy Questionnaire
ACC: American College of Cardiology.
