# Supplementary material for: Efficacy and safety of sacubitril/valsartan on heart failure with preserved ejection fraction: A meta-analysis of randomized controlled trials
Source: Front Cardiovasc Med. 2022 Sep 8;9:897423. doi: 10.3389/fcvm.2022.897423 (PMC9492872; doi:10.3389/fcvm.2022.897423)
Supplement: Supplementary file 1 [file Data_Sheet_1.docx]

| Supplemental Table 1: Baseline characteristics of RCTs | | | | | | |  |
| --- | --- | --- | --- | --- | --- | --- | --- |
|  | PARALLAX-HF Pieske 2021 | | PARAGON-HF Solomon 2019 | | PARAMOUNT Solomon 2012 | |  |
|  |  |  |  |  |  |  |  |
| Group | Sac/Val (n=1281) | IMT (n=1285) | Sac/Val (n=2407) | valsartan (n=2389) | Sac/Val (n=149) | valsartan (n=152) |  |
| Follow-up Duration | 12 weeks | | 8 months | | 36 weeks | |  |
| Age, y | 72.9±8.4 | 72.4±8.6 | 72.7±8.3 | 72.8±8.5 | 70.9±9.4 | 71.2±8.9 |  |
| Female (%) | 638(49.8) | 627(48.8) | 1241 (51.6) | 1238 (51.8) | 85 (57) | 85 (56) |  |
| White race (%) | 1112 (86.8) | 1117 (86.9) | 1963 (81.6) | 1944 (81.4) | NA | NA |  |
| NYHA class (%) |  |  |  |  |  |  |  |
| I | 1 (0.1) | 4 (0.3) | 73 (3.0) | 64 (2.7) | 1 (1) | 1 (1) |  |
| II | 858 (67) | 876(68.2) | 1866 (77.5) | 1840 (77.0) | 120 (81) | 119 (78) |  |
| III | 416(32.5) | 401(31.2) | 458 (19.0) | 474 (19.8) | 28 (19) | 32 (21) |  |
| IV | 5（0.4） | 4（0.3） | 8 (0.3) | 11 (0.5) |  |  |  |
| LVEF (%) | 56.7±8.3 | 56.2±8.0 | 57.6±7.8 | 57.5±8.0 | 58±7.3 | 58±8.1 |  |
| Heart rate, beats/min | NA | NA | 70.6±12.3 | 70.3±12.2 | 69±12 | 70±14 |  |
| Systolic blood pressure, mmHg | 132.6±13.9 | 134.2±14.5 | 130.5±15.6 | 130.6±15.3 | 137.1±11.2* | 135.7±14.2* |  |
| Body mass index | 30.6±5.0 | 30.5±4.8 | 30.2±4.9 | 30.3±5.1 | 30.1±5.5 | 29.8±6.1 |  |
| Scr, mg/dl |  |  | 1.1±0.3 | 1.1±0.3 |  |  |  |
| GFR, ml/min/1.73m^2^ | 62.5±20.2 | 62.7±19.6 | 63±19 | 62±19 | 67±19·4 | 64 ±21·3 |  |
| Potassium, mmol/L | NA | NA | NA | NA | NA | NA |  |
| Medications at baseline (%) |  |  |  |  |  |  |  |
| ACE inhibitors | 1115(87.1) | 1124(87.5) | 2074 (86.2) | 2065 (86.4) | 83 (56) | 80 (53) |  |
| ARBs |  |  |  |  | 57 (38) | 62 (41) |  |
| Diuretics | 1277 (99.8) | 1282(99.8) | 2294 (95.3) | 2291 (95.9) | 149 (100) | 152 (100) |  |
| Beta-blockers | 1071 (83.7) | 1066(83) | 1922 (79.9) | 1899 (79.5) | 117 (79) | 121 (80) |  |
| Aldosterone antagonists | 419(32.7) | 392(30.5) | 592 (24.6) | 647 (27.1) | 28 (19) | 35 (23) |  |

Abbreviations: Sac/Val, sacubitril-valsartan; NYHA, New York Heart Association; LVEF, left ventricular ejection fraction; Scr, serum creatinine; GFR, glomerular filtration rate; ACE, angiotensin-converting enzyme; ARBs, angiotensin receptor blockers; RCTs, randomized controlled trials.

*The sample mean and standard deviation was estimated from the sample size, median, interquartile range through the special website (<http://www.math.hkbu.edu.hk/~tongt/papers/median2mean.html>).

1. Pieske B, Wachter R, Shah SJ, Baldridge A, Szeczoedy P, Ibram G, Shi V, Zhao Z, Cowie MR; PARALLAX Investigators and Committee members. Effect of Sacubitril/Valsartan vs Standard Medical Therapies on Plasma NT-proBNP Concentration and Submaximal Exercise Capacity in Patients With Heart Failure and Preserved Ejection Fraction: The PARALLAX Randomized Clinical Trial. JAMA. 2021 Nov 16;326(19):1919-1929.

2. Solomon SD, McMurray JJV, Anand IS, Ge J, Lam CSP, Maggioni AP, Martinez F, Packer M, Pfeffer MA, Pieske B, Redfield MM, Rouleau JL, van Veldhuisen DJ, Zannad F, Zile MR, Desai AS, Claggett B, Jhund PS, Boytsov SA, Comin-Colet J, Cleland J, Dungen HD, Goncalvesova E, Katova T, Kerr Saraiva JF, Lelonek M, Merkely B, Senni M, Shah SJ, Zhou J, Rizkala AR, Gong J, Shi VC and Lefkowitz MP. Angiotensin-Neprilysin Inhibition in Heart Failure with Preserved Ejection Fraction. N Engl J Med. 2019;381:1609-1620.

3. Solomon SD, Zile M, Pieske B, Voors A, Shah A, Kraigher-Krainer E, Shi V, Bransford T, Takeuchi M, Gong J, Lefkowitz M, Packer M and McMurray JJ. The angiotensin receptor neprilysin inhibitor LCZ696 in heart failure with preserved ejection fraction: a phase 2 double-blind randomised controlled trial. Lancet. 2012;380:1387-95.
