# Supplementary figures and images for: Efficacy and safety of sacubitril/valsartan on heart failure with preserved ejection fraction: A meta-analysis of randomized controlled trials
Source: Front Cardiovasc Med. 2022 Sep 8;9:897423. doi: 10.3389/fcvm.2022.897423 (PMC9492872; doi:10.3389/fcvm.2022.897423)

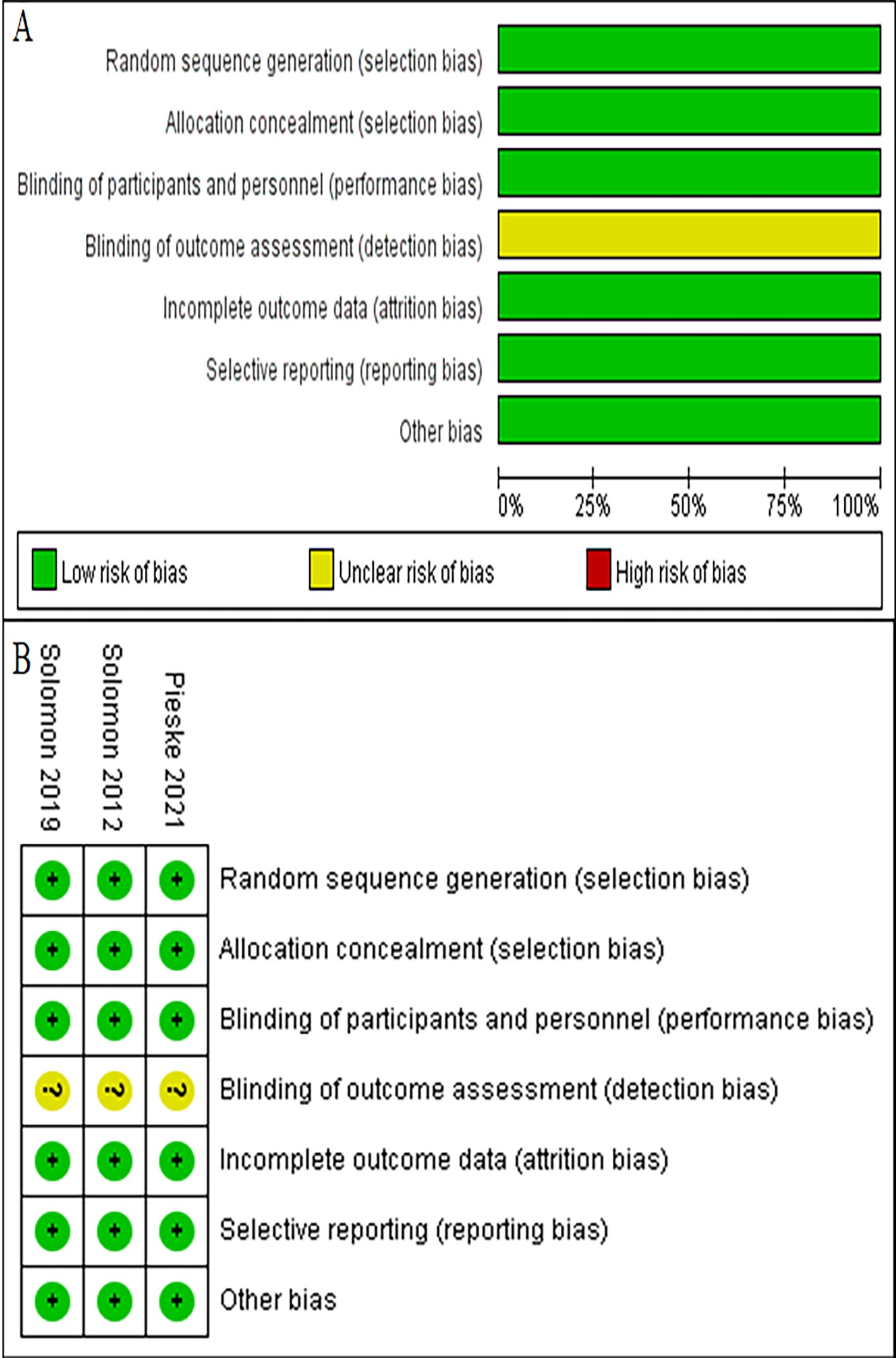

Supplement: Supplementary file 2 [file Data_Sheet_1.ZIP › Figure S1.jpg]
